# Cut-Off Levels of Anti-Mullerian Hormone for The Prediction of Ovarian Response, *In Vitro* Fertilization Outcome and Ovarian Hyperstimulation Syndrome 

**DOI:** 10.22074/ijfs.2015.4236

**Published:** 2015-07-27

**Authors:** Ali Salmassi, Liselotte Mettler, Jurgen Hedderich, Walter Jonat, Anupama Deenadayal, Soeren von Otte, Christel Eckmann-Scholz, Andreas Gerd Schmutzler

**Affiliations:** 1 Department of Gynaecology and Obstetrics, Center of Reproductive Medicine, University Hospitals Schleswig-Holstein, Campus Kiel, Kiel, Germany; 2 Institute of Medical Informatics and Statistics, University of Kiel, Kiel, Germany

**Keywords:** Anti Mullerian Hormone, Ovarian Hyperstimulation Syndrome, Cut-Off Levels, Pregnancy Rate

## Abstract

**Background:**

Evaluation of anti-mullerian hormone (AMH) cut-off levels in as- sisted reproductive technology (ART) as predictive factor for individualization of stimulation protocols and to avoid ovarian hyperstimulation syndrome (OHSS).

**Materials and Methods:**

In a retrospective study, 177 infertile patients were as- sessed for AMH in serum and follicular fluid (FF) on the day of follicular puncture (FP), between 2012 and 2013 in Kiel, Germany. AMH levels and pregnancy rates were compared between low, moderate and high responders and cut-off levels of low and high responders. AMH cut-off levels in pathological cases were evaluated in analysis 1 (OHSS) and in analysis 2 [polycystic ovarian syndrome, (PCOS)] and compared in analysis 3 to normal endocrinological parameters.

**Results:**

AMH levels in FF were higher than in serum (P<0.001). AMH levels in serum and FF increased from low through moderate to high responders (P<0.001). Pregnancy rates were 14.7, 23.3 and 44.9% (P=0.009), respectively. AMH cut-off level for poor responders was 0.61 ng/ml in serum with a pregnancy rate of 13.8 and 37.1% for below and above of this level, respectively. For FF, it was 1.43 ng/ml. AMH levels in analysis 1 and 2 were significantly higher than in analysis 3 (P=0.001). AMH cut-off level for OHSS was 1.5 ng/ml in serum with OHSS rates of 80.8 and 19.2 % for above and below of the level, respectively. For FF, it was 2.7 ng/ml. PCOS patients had an AMH cut-off level of 3.9 ng/ml in serum and 6.8 ng/ml in FF, resulting in a PCOS rate of 100% above this level.

**Conclusion:**

AMH levels can help to assess ovarian response potential and guide ovarian stimulation while avoiding OHSS.

## Introduction

Anti-mullerian hormone (AMH) in the female ovary is produced by granulosa cells of pre-antral and antral follicles (1). The main physiological role of AMH in the ovary is limited to the inhibition of the early stages of follicular development (2-6).

According to current literature, AMH appears to be a promising and reliable marker of the number of small antral follicles, with essentially constant levels across the cycle and a superior intercycle reproducibility compared with that of follicle stimulating hormone (FSH) and early antral follicle count (7-13). Hence, it has the potential to determine the plan of ovarian stimulation in an assisted reproductive technology (ART) cycle. As AMH levels steadily decline with age from adulthood toward menopause, AMH is a promising parameter for early detection of reduced ovarian reserve as well as ovarian dysfunction (12, 14-17). Patients with ovarian hyperstimulation syndrome (OHSS) have high serum levels of AMH prior to controlled ovarian stimulation (COS). For these patients, the COS protocol can be individualized to suit their requirements (6, 18, 19). The specific risk factors for OHSS include young age, low body mass index (BMI) and signs of polycystic ovarian syndrome (PCOS) (20-22). PCOS affects 5-20% of women of reproductive age and is the primary cause of anovulatory infertility, with an increased number of antral follicles and a resulting rise of AMH (11, 23, 24).

Serum AMH levels in women with PCOS are higher than in ovulatory women. However, urgently needed cut-off levels of AMH in patients with endocrinological risk factors, such as PCOS and the hormonally induced overreaction of the ovarian response as in OHSS, are still missing to support the clinical decision (11, 25-28). In this retrospective study, we attempted to assess and compare the mean and cut-off levels of AMH in serum and follicular fluid (FF) on the day of oocyte retrieval in response to ovarian stimulation with recombinant follicle stimulating hormone (rFSH) and their relation to pregnancy rates. Furthermore, we planned to identify the AMH mean and cut-off levels in serum and FF in patients with endocrinological risk factors and pathological factors, such as OHSS or PCOS, and compare these to the values of patients without these risk factors.

## Materials and Methods

### Patients

For this retrospective study, we collected serum and follicular fluid for the first or second treatment cycle of 177 patients undergoing *in vitro* fertilization (IVF) and intracytoplasmic sperm injection (ICSI) (between 2012 and 2013) in Kiel. Serum and FF were collected on the day of follicular puncture (FP). The age of the patients ranged from 20 to 42 years, median 33 years, and the size of the leading follicle on the day of follicular puncture measured between 19 and 24 mm. The FF for AMH analysis was only aspirated from the first dominant follicle. All 177 patients presented with tubal or male factor infertility.

The patients were analysed repeatedly and pathological cases were evaluated in analysis 1 (OHSS) and analysis 2 (PCOS) and compared to normal endocrinological parameters (analysis 3). All patients treated (n=177) revealed in their initial endocrine check, on days 3-5 of the cycle, an AMH value >0.5 ng/ml and a FSH value <8 IU/ ml. For analysis 2 and 3, we specially collected the cases between 2012 and 2013.

AMH levels of all patients were analysed for:

Correlation between serum and FF with respect to AMH levels and correlation among serum AMH, serum estradiol (E_2_), the number of follicles, injected dose of rFSH and age of patients.
Evaluation of mean AMH and cut-off levels in serum and FF in response to ovarian stimulation with rFSH in low (n=41), moderate (n=66) and high (n=70) responders and pregnancy rates.

In analysis 1: AMH cut-off levels were evaluated for patients with hormonally induced overreaction of the ovary, such as OHSS (n=26). In analysis 2: AMH levels were evaluated for patients with the endocrinological risk factor, PCOS (n=30) and in versus analysis 3: patients with normal endocrinological parameters (n=121).

Analysis 1 consisted of patients with peak serum levels of E_2_>3000 pg/ml on the day of ovulation induction and with signs or symptoms consistent with OHSS, such as ultrasonographic evidence of ascites and increased ovarian size of 8-12 cm, abdominal bloating and pain, or considerable weight gain (18, 29).

Analysis 2 consisted of patients with signs of PCOS who were diagnosed by the Rotterdam criteria with two of the following three manifestations: irregular or absent ovulation, elevated levels of androgenic hormones and/or enlarged ovaries containing at least 12 follicles each (30).

### Ovarian stimulation

rFSH (Gonal F, Merck, Serono, Munich, Germany) after down-regulation with gonadotropinreleasing hormone agonist (GnRH-a) (Synarela, Pharmacia, Erlangen, Germany) was used in the long protocol. The FSH doses were adapted according to the following criteria: age of patient, number of antral follicles, AMH, basal FSH and patient’s diagnosis. Monitoring of follicle development by real-time ultrasound scans and serum E_2_ levels was performed from day 6 of stimulation every two to three days until the day of human chorionic gonadotropin (hCG) application. Ideally, once the leading follicle measured >16 mm in diameter and the 17 β - E_2_ level had adequately increased ideally to around 3000 pg/ml in serum, 6500 IU of hCG were administered subcutaneously. The number of follicles was determined on the day of ovulation induction (n=10.2 ± 6.4). Progesterone (Pr) levels were measured parallel to E_2_ and luteinizing hormone (LH). Follicles were aspirated 36 hours after administration of hCG. After embryo transfer (ET), the patients were treated with Pr vaginally (Utrogest, 600 mg daily, Dr. Kade/ Besins, Berlin, Germany) for luteal support until confirmation of pregnancy by beta-hCG (β-hCG) determination, 14 days after ET.

In response to ovarian stimulation, the patients were sub-grouped as low, moderate and high responders, according to a scoring system based on the total injected dose of rFSH [± standard deviation (SD)] up to the day of hCG injection, the increase in E_2_ levels, the age of patients and the number of follicles (low ≤7, moderate=8-14 and high ≥15) on the day of ovulation induction (31- 33). Only the clinically continuing pregnancy rates per ET were evaluated.

### Biochemical analyses

#### Anti-mullerian hormone assay in serum

Blood and FF were taken from all patients undergoing IVF or ICSI and ET on the day of follicular puncture, processed by being centrifuged for 10 minutes at 350×g and 5˚C, shock-frozen and kept at −80˚C. After pick-up of the oocytes, the FF samples underwent the same procedures as the blood. AMH levels in serum and FF were measured in duplicate by a solid-phase enzyme-linked immunosorbent assay (ELISA) using an AMH kit (AMH Gen II Assay, Immunotech, Beckman Coulter Company, Kiel, Germany). This assay uses the quantitative sandwich enzyme immunoassay technique. The AMH precision from manufacture was CV=3.2-12.3% for intra-assay and CV=5.8-14.2% for inter-assay. Only those cases in which both FF and serum could be collected simultaneously on the day of oocyte retrieval were included in this study.

#### Estradiol assay in serum

E_2_ levels were measured by a solid phase, competitive chemiluminescent enzyme immunoassay with the Immulite 2000 auto system (DPC-Biermann; Siemens, Bad Nauheim, Germany) within the range of 0–2000 pg/ml for E_2_ (sensitivity 15 pg/ml).

### Statistical evaluation

All statistical analyses were performed using the Statistical Package for the Social Sciences (SPSS, SPSS Inc., Chicago, IL, USA) version 20. Based on the Kolmogorov-Smirnov test, normal distribution for most of the parameters could not be assumed. Therefore, in descriptive statistics median values and interquartile ranges (IQR) were given additionally to means and SDs. Nonparametric test procedures were used for statistical evaluation of the study data.

We performed a Kruskal-Wallis test to analyse differences in AMH levels between patients with low, moderate and high response to ovarian stimulation. In the case of any significance, pairwise comparisons between different groups were performed with the U test in an exploratory intention. The differences in pregnancy rates between low, moderate and high response patients were analysed according to a chi-squared test. Association between serum and FF with respect to AMH and between AMH and E_2_ inserum was measured and tested by Spearman rank correlation coefficients (r_s_).

A P value of P<0.05 was considered to be statistically significant throughout the study. To evaluate an AMH cut-off value in serum and in FF related to patients with (low/moderate and high) response for ovarian stimulation as well as to patient’s risk for OHSS or PCOS, a receiver operating characteristic (ROC) analysis was performed to achieve minimal false positive and false negative results. The U test was also used to evaluate the hypotheses of differentiation [area under the curve (AUC) >0.5, i.e. low responder vs. moderate and high responder].

### Ethical considerations

An informed consent was obtained from all patients in the university IVF program. Under the stipulations of the Universitaetsklinikum Schleswig-Holstein (UKSH), Kiel Institutional Review Board (IRB), an approval had not to be obtained for a retrospective observational study.

## Results

### AMH levels in serum and FF on the day of oocyte retrieval

**Fig.1:**
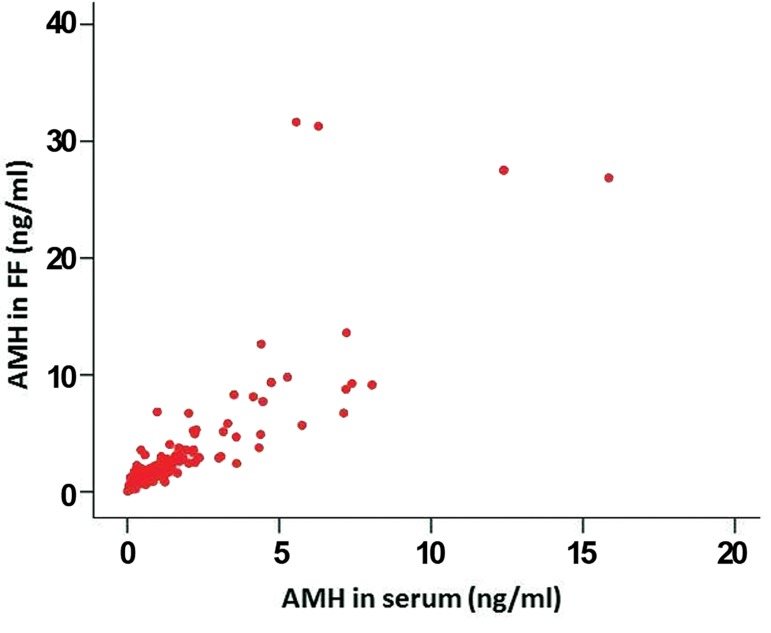
Correlation between AMH levels (n=177) in serum and in FF on the day of oocyte retrieval (r_s_=0.88, P<0.001).

The median AMH level in FF, 2.2 ng/ml (1.32-3.6) on the day of oocyte retrieval, was significantly higher than that in serum, 1.14 ng/ ml (0.52-2.17, P<0.001). On the basis of nonnormal distributed values of AMH levels in serum and FF, we found a positive correlation (Spearmann-Rho rs= 0.88, P<0.001, Fig.1). Additionally, we observed a significant positive correlation between some characteristic clinical parameters and AMH in serum or in FF on the day of FP as follows: E_2_ (r_s_=0.43), number of follicles (r_s_-=-0.71), number of total retrieved oocytes and oocytes in MII (r_s_=0.34), number of fertilized oocytes (r_s_=0.32) and a significant inversed correlation with regard to age (r_s_=-0.55), total injected dose (r_s_=-0.63) and BMI (r_s_=- 0.21). The median AMH level, 1.76 ng/ml (1.0- 3.73) in serum and 2.9 ng/ml (1.77- 6.75) in FF, of patients who became pregnant was significantly higher than in those who did not become pregnant, 1.0 ng/ml (0.4-1.65) in serum and 1.8 ng/ml (1.0-2.9) in FF (P<0.004 and P<0.01, respectively).

### Evaluation of AMH levels in response to ovarian stimulation and pregnancy rate

**Fig.2:**
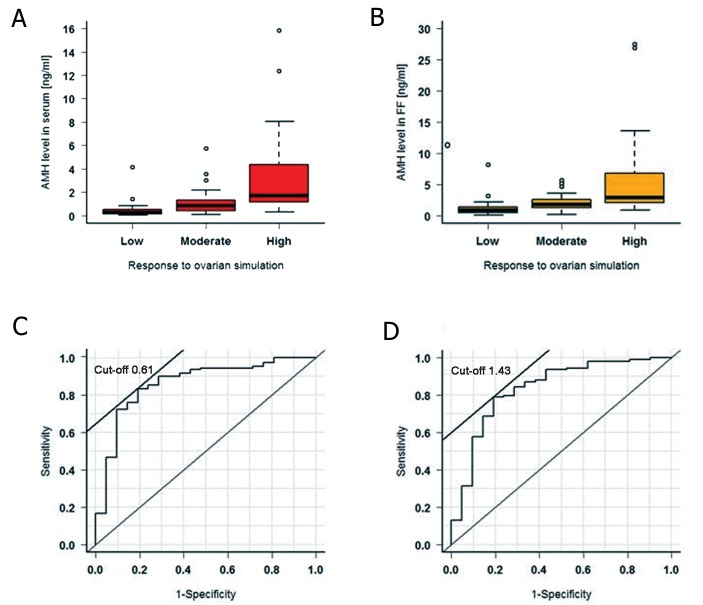
Relationship between anti-mullerian hormone (AMH, n=177) levels in serum (A), in follicular fluid (FF) (B) and low, moderate and high response to ovarian stimulation (C-D). Receiver operating characteristic (ROC) area for low responder patients versus moderate and high responders in serum (C) and in FF (D) on the day of FP (Follicular Puncture). The differences between low, moderate, and high responders were significant (P<0.001). See table 1 for paired comparisons according to the Mann-Whitney U test.

The AMH levels in serum and in FF of patients with ET and their response to ovarian stimulation with gonadotropins are summarised in table 1 and figure 2A, B.

AMH levels in serum and in FF increased significantly from patients with low response through moderate and reached a maximum in patients with high response. Similar to AMH, E_2_, number of oocytes and pregnancy rate increased from low to high responders. In contrast, age and the injected total rFSH-dose showed a significant decrease.

The differences in AMH, total injected dose, E_2_ concentrations, number of oocytes and pregnancy rate in serum and FF among the three groups were statistically significant (P<0.001). Paired comparisons of AMH levels and all other clinical parameters in serum and in FF were significant in all pairs (Table 1). The total pregnancy rate was 31.6% related to ET.

**Table 1:** 177 patients sub-grouped according to their response to ovarian stimulation with rFSH as low, moderate and high responders related to AMH values and pregnancy rates


Response	Low (n=41)	Moderate (n=66)	High (n=70)	P value	*P
Variables	Mean ± SD Median (IQR)	Mean ± SD Median (IQR)	Mean ± SD Median (IQR)		

Age (Y)	38 ± 4.1	35.7± 4.2	33.6 ± 4.4	0.001	0.07 ^b^,0.001 ^c^
	39 (35-41)	37 (33-39)	33 (30-37)		0.011 ^d^
Total mean level of rFSH (iu/ml)	4901 ± 1452	3204 ± 1311	2460± 977	<0.001	<0.00 ^b^^,^^c^
	5400 (3525-6000)	2887 (2250-4200)	2250(1762-3362)		0.005 ^d^
E2(pg/ml)	527 ± 305	1021 ± 451	1285± 470	<0.001	<0.001 ^b^^,^^c^
	590 (203-732)	954 (774-1269)	1297(994-1594)		0.002 ^d^
Number of oocytes	3.4 ± 1.5	7.5 ± 2.9	12.3 ± 5.5	<0.001	<0.001 ^b^^,^^c^^,^^d^
	3 (2.5-4)	8 (5-10)	12 (8-16)		
AMH in serum (ng/ml)	0.54 ± 0.46	1.1 ± 1.0	3.03 ± 2.6	<0.001	0.001 ^b^
	0.3 (0.13-0.54)	0.84(0.42-1.32)	1.7 (1.3-4.36)		<0.001 ^c^^,^^d^
AMH in FF (ng/ml)	1.3 ± 1.12	2.01± 1.18	5.94 ± 4.3	<0.001	0.001 ^b^
	0.87 (0.45-1.41)	1.75(1.24-2.56)	2.9 (2.07-6.78)		<0.001 ^c^^,^^d^
Pregnancy rate	14.7%	23.3%	44.9%	0.009^a^	


rFSH; Recombinant follicle stimulating hormone, E_2_; Estradiol, FF; Follicular fluid, AMH; Anti-mullerian hormone, IQR; Interquartile range, P; Kruskal-Wallis test, , *P; Pair wise comparisons between sub-groups, ^a^; Chi-square test, ^b^; Low/moderate, ^c^; Low/high and ^d^; Moderate/ high. Values are mean ± SD unless otherwise noted.

### Evaluation of AMH cut-off levels in serum and FF in response to ovarian stimulation

#### a. Low responder

Figure 2 (c+d) shows the typical receiver ROC AUC for AMH, indicating low responders versus moderate and high responders (n=136) in serum (c) and in FF (d) on the day of FP.

We found an AMH cut-off level (based on best sensitivity and best specificity) in serum of 0.61 ng/ml and a pregnancy rate of 13.8 (n=6/44) below and 37.6% (n=50/133) above this cut-off level (Table 2). AMH cut-off level in FF was 1.43 ng/ml and revealed a pregnancy rate of 12.5 (n=5/40) below and 38.3% (n=51/133) above this cut-off level.

#### b. High responder

AMH cut-off level (based on best sensitivity and best specificity) in serum was 1.03 ng/ml for high responders versus low and moderate responders, and resulted in a pregnancy rate of 21.1 (n=16/76) below and 38.6% (n=39/101) above this cut-off level (Table 2).

The AMH cut-off level in FF was 2.23 ng/ml with a pregnancy rate of 21.5 (n=17/79) below and 39.8% (n=39/98) above this cut-off level.

**Table 2:** Calculated cut-off levels of AMH predicting low and high responders in serum and in FF, sensitivity (true positive rate), specificity (true negative rate) and pregnancy rate


AMH	ROC-AUC	P value	Sensitivity %	Specificity %	Cut-off ng/ml	Pregnancyrate %≤ cut-off	Pregnancyrate %> cut-off

Serum ^a^	0.86	<0.001	83.5	81	0.61	13.8	37.6
FF ^a^	0.84	<0.001	78.9	81	1.43	12.5	38.3
Serum ^b^	0.83	<0.001	87.7	71.6	1.03	21.1	38.6
FF ^b^	0.80	<0.001	73.9	75.4	2.23	21.5	39.8


AMH; Anti-mullerian hormone, FF; Follicular fluid, ROC-AUC; Receiver operating characteristic-area under curve, ^a^; Low versus both moderate and high and ^b^; High versus both low and moderate.

### Comparisons of AMH levels between patients with OHSS, PCOS and patients with normal endocrinological parameters

**Fig.3:**
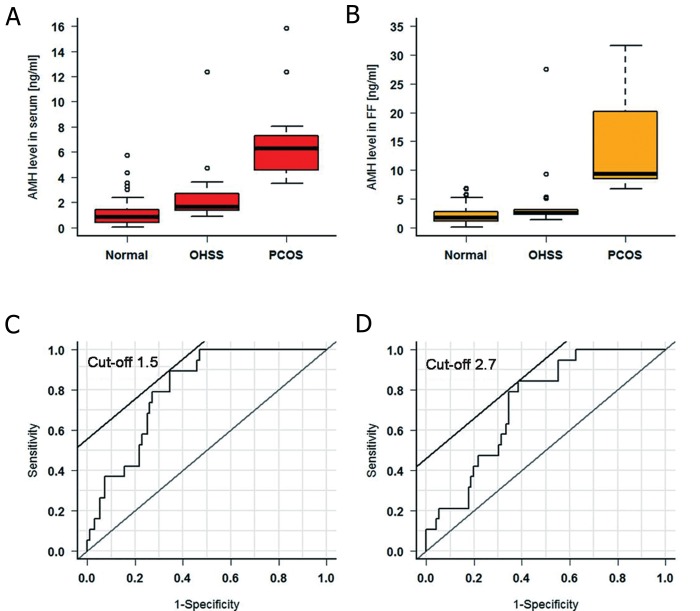
Comparison of anti-mullerian hormone (AMH) levels in serum (A), in follicular fluid (FF) (B) between patients with normal endocrinological parameters and patients with ovarian hyperstimulation syndrome (OHSS) or polycystic ovarian syndrome (PCOS) (C-D). Typical receiver operating characteristic (ROC) for AMH level in serum (C) and in FF (D) for OHSS patients versus normal patients.

Patients with OHSS (analysis 1) or PCOS (analysis 2) revealed a significantly lower median level of total injected dose of rFSH and a higher count of follicles, suggesting higher level of E_2_ (on the day of hCG injection) than patients with normal endocrinological parameters (analysis 3) (Table 3). A significantly higher number of oocytes was obtained on the day of FP only from patients with OHSS. As seen in table 3 and figure 3a-b, patients with normal endocrinological parameters revealed the lowest levels of AMH and patients with an endocrinological risk of PCOS showed the highest mean levels of AMH for serum and for FF.

According to the Mann-Whitney test, paired comparisons of AMH levels in serum and in FF between sub-groups were significant: OHSS/normal (P=0.009) and PCOS/normal (P<0.001).

**Table 3:** Comparison between patients with normal endocrinological parameters and patients with OHSS or PCOS


	Normal (n=121) Mean ± SD Median (IQR)	OHSS(n=26)Mean ± SDMedian (IQR)	PCOS (n=30) Mean ± SD Median (IQR)	P value	*P

Total injected dose (IU/ml)	3394± 1480	2477 ± 725	2084± 1194	<0.001	0.018 ^a^
	3000(2325-4500)	2100 (1912-2850)	1575(1200-3350)		0.002 ^b^
Number of follicles	14 ± 6.4	23.5± 6.5	27.3 ± 9.5	<0.001	<0.001 ^a^
	13.5 (8-19)	24 (18-28)	26 (23-35)		<0.001 ^b^
Number of oocytes	8.1± 4.5	15.9± 6.2	8.6 ± 5.8	<0.001	<0.001 ^a^
	8 (4.2-11)	14 (10-21)	8 (3-12)		NS ^b^
E2(pg/ml)	1844± 536	2907 ± 1430	2759± 1143	<0.01	<0.001 ^a^
	1795(1275-2165)	3063(1911-3371)	2561(1860-3094)		<0.001 ^b^
Mean AMH Serum (ng/ml)	1.2± 1.1	2.52± 2.1	7.2 ± 3.5	0.001	<0.001 ^a^
	0.85 (0.4-1.4)	1.64(1.38-3.1)	6.3 (4.4-7.4)		<0.001 ^b^
Mean AMH FF (ng/ml)	2.1± 1.4	4.4 ± 3.1	14.5 ± 8.5	0.014	0.009 ^a^
	1.7(1.05-2.8)	2.52(2.27-3.1)	9.35 (8.3-26.8)		<0.001 ^b^


OHSS; Ovarian hyperstimulation syndrome, PCOS; Polycystic ovarian syndrome, E_2_; Estradiol, AMH; Anti-mullerian hormone, FF; Follicular fluid, IQR; Interquartile range, ^a^; OHSS/normal and ^b^; PCOS/normal. Total injected dose of rFSH up to the day of hCG injection, E_2_ level on the day of hCG injection. The differences between these 3 groups were analysed by the Kruskal-Wallis test (P) and paired comparisons (*P) by the Mann-Whitney U test.

### Evaluation of AMH cut-off levels in serum and in FF in patients with OHSS or PCOS

We found an AMH cut-off level (based on best sensitivity and best specificity) in serum of 1.5 ng/ ml, and in FF of 2.7 ng/ml (Table 4, Fig.3C, D), between patients with OHSS and those without, occasioning an OHSS rate of 19.2 (5/26) below and 80.8% (21/26) above these levels. Additionally, we found that 90% of patients with OHSS had an AMH level below 4 ng/ml in serum.

The AMH cut-off level in serum for PCOS in comparison to normal patients was 3.9 ng/ml, with a high sensitivity and specificity, resulting in a PCOS rate of 100% above this level.

In FF, the cut-off level was 6.8 ng/ml, also resulting in a PCOS rate of 100% above this level.

**Table 4:** Calculated cut-off level of AMH predicting OHSS and PCOS in serum or in FF, specificity (true negative rate), sensitivity (true positive rate)


		ROC-AUC	P value	Sensitivity(%)	Specificity(%)	Cut-offlevel (ng/ml)	Risk of ≤cut-off level%	Risk of >cut-off level%

OHSS (n=26)	Serum	0.79	<0.001	79	73	1.5	21	79
	FF	0.73	0.004	79	66	2.7	21	79
PCOS (n=30)	Serum	0.98	<0.001	93	97	3.9	0	100
	FF	0.97	<0.001	93	98	6.8	0	100


OHSS; Ovarian hyperstimulation syndrome, PCOS; Polycystic ovarian syndrome, AMH; Anti-mullerian hormone, FF; Follicular fluid and ROC-AUC; Receiver operating characteristic-area under curve.

## Discussion

In this study, we analysed for the first time simultaneously the AMH levels in serum and in FF on the day of oocyte retrieval and compared them between patients with OHSS and PCOS with nomal endocrinological parameters.

Our results showed that AMH concentrations in FF are significantly higher than in serum, as found in the study of Takahashi et al. (34). This implies an intrafollicular production and a potential autocrine or paracrine role of AMH within the follicular environment. AMH is expressed only in the ovarian granulosa cells of primary follicles and plays an important role in ovarian function, especially in follicle differentiation, development and selection (1, 5, 10, 34). The mean AMH levels in serum and in FF of patients who became pregnant were significantly higher than in those who did not become pregnant. Some studies have also reported that AMH can predict pregnancy (7, 13, 34, 35). AMH levels in serum and in FF on the day of FP increased significantly with the decreasing age of patient and with an increasing follicle count on the day of hCG injection. Similar results in serum on day three of the cycle have shown that the loss of follicles with increasing female age is variable and that the chronological age of the ovary does not always reflect its biological and reproductive age (14, 36).

With regard to the response to ovarian stimulation with rFSH, AMH levels increased in serum and in FF significantly from low through moderate to high responders with a respective pregnancy rate of 14.7, 23.3 and 44.9 %. Thus, AMH levels in serum and in FF may reflect successful stimulation and ample follicle maturation.

The characterisation of AMH as a sensitive marker for poor ovarian reserve (13, 18, 37-39) was further evaluated by our AMH cut-off levels for low and high responders with a high sensitivity and specificity. Our evaluated cut-off levels for AMH are in the range proposed by the European Society of Human Reproduction and Embryology (ESHRE) consensus meeting (cut-off level from 0.5-1.1 ng/ml) (33). With these cut-off levels and a very high accurancy for AMH (AUC=0.86 in serum), there were distinct significant differences in the pregnancy rate between low responders (13.8 below versus 37.1% above) and high responders (21.7 below versus 39.8% above). Broer et al. (40) also found similar levels of AUC=0.78 and for AMH, AUC predicting poor response. In accordance with these cut-off levels, patients can be counselled regarding the expected outcome of ovarian response, number of follicles and the anticipated cost of ovarian stimulation drugs, thereby reducing the emotional and financial burden of cycle cancellation. Fleming et al. (35) also reported that AMH is one of the best accepted markers of ovarian reserve and a strong marker for response to stimulation.

We found a significant inverse correlation between AMH level and BMI, which is in good agreement with other authors (41, 42). OHSS represents one of the most serious complications subsequent to COS and can be life-threatening (43). The mean AMH levels in serum and in FF were significantly higher in patients with OHSS and PCOS than in normal patients.

Other authors predict a basal AMH cut-off level of around 3.36 ng/ml for OHSS with a high sensitivity and specificity (22, 43, 44), indicating high rates of OHSS above this level. Our evaluated AMH cut-off level for OHSS patients of 1.5 ng/ ml in serum (patients with PCOS excluded) corresponds well with the evaluated level of 1.6 ng/ ml found by Ebner et al. (45). This level revealed a high sensitivity, specificity and high accuracy of AUC, resulting in significantly distinct differences in the OHSS rate of 19.2 below versus 80.8% above these levels. About 90% of patients with OHSS had a serum AMH below 4 ng/ml, suggesting that clinicians have become cautious in stimulating patients with an AMH above 4 ng/ml. It has been reported that AMH levels in serum decline gradually during controlled ovarian hyperstimulation (COH), whereas other hormones, such as E_2_, inhibin A, inhibin B and Pr, increase (46, 47). It has been suggested that this reflects the reduction in the number of small antral follicles parallel to the increase in the number of larger ones. This could also indicate that basal AMH levels and AMH levels on the day of FP differ considerably and should be investigated further.

The assessed AMH cut-off level for PCOS patients in serum and in FF showed a high sensitivity and specificity resulting in all patients, showing an AMH value above this level.

Supporting our results, other authors have also reported a relatively high specificity of 92% but a low sensitivity of 67% of AMH as a diagnostic marker for PCOS (6, 11). On this basis, it has been proposed that in situations where accurate ultrasound data are not available, AMH could be used in addition to the follicle count as a diagnostic criterion for PCOS (11, 48).

## Conclusion

AMH levels are a sensitive parameter for the prediction of response to ovarian stimulation with gonadotropins. The levels can be used as a tool for pre-stimulation patient counselling regarding the expected ovarian response (poor, moderate and high) and outcome (pregnancy rate, OHSS and cycle cancelation). Additionally, it can be used as a marker for PCOS. Its application for guiding appropriate stimulation protocols can be used to avoid OHSS.
